# Ascorbic acid ameliorates renal injury in a murine model of contrast-induced nephropathy

**DOI:** 10.1186/s12882-017-0498-5

**Published:** 2017-03-24

**Authors:** K. Rollins, A. Noorani, L. Janeckova, T. Jones, M. Griffiths, M. P. Baker, J. R. Boyle

**Affiliations:** 1 National Institute for Health Research Nottingham Digestive Diseases Biomedical Research Unit, Nottingham University Hospitals NHS Trust, Queen’s Medical Centre, Nottingham, UK; 2 Antitope Ltd, Babraham Research Campus, Cambridge, UK; 3 Department of Histopathology, Addenbrookes Hospital, Cambridge, UK; 4 Cambridge Vascular Unit, Addenbrookes Hospital, Cambridge, UK

**Keywords:** Contrast-induced nephropathy, Ascorbic acid, Nephroprotection, Histopathology, Immunohistochemistry, Endovascular procedures

## Abstract

**Background:**

Contrast induced nephropathy (CIN) is the commonest cause of iatrogenic renal injury and its incidence has increased with the advent of complex endovascular procedures. Evidence suggests that ascorbic acid (AA) has a nephroprotective effect in percutaneous coronary interventions when contrast media are used. A variety of biomarkers (NGAL, NGAL:creatinine, mononuclear cell infiltration, apoptosis and RBP-4) in both the urine and kidney were assayed using a mouse model of CIN in order to determine whether AA can reduce the incidence and/or severity of renal injury.

**Methods:**

Twenty-four BALB/c mice were divided into 4 groups. Three groups were exposed to high doses of contrast media (omnipaque) in a well-established model of CIN, and then treated with low or high dose AA or placebo (saline). CIN severity was determined by measurement of urinary neutrophil gelatinase-associated lipocalin (NGAL):creatinine at specific time intervals. Histological analysis was performed to determine the level of mononuclear inflammatory infiltration as well as immunohistochemistry to determine apoptosis in the glomeruli by staining for activated caspase-3 and DNA nicking (TUNEL assays). Reverse transcriptase PCR (rtPCR) of mRNA transcripts prepared from mRNA extracted from mouse kidneys was also performed for both lipocalin-2 (Lcn2) encoding NGAL and retinol binding protein-6 (RBP4) genes. NGAL protein expression was also confirmed by ELISA analysis of kidney lysates.

**Results:**

Urinary NGAL:creatinine ratio was significantly lower at 48 h with a 44% and 62% (204.3μg/mmol versus 533.6μg/mmol, *p* = 0.049) reduction in the low and high dose AA groups, respectively. The reduced urinary NGAL:creatinine ratio remained low throughout the time period assessed (up to 96 h) in the high dose AA group. In support of the urinary analysis ELISA analysis of NGAL in kidney lysates also showed a 57% reduction (12,576 ng/ml versus 29,393 ng/ml) reduction in the low dose AA group. Immunohistochemistry for apoptosis demonstrated decreased TUNEL and caspase-3 expression in both low and high dose AA groups.

**Conclusions:**

Ascorbic acid reduced the frequency and severity of renal injury in this murine model of CIN. Further work is required to establish whether AA can reduce the incidence of CIN in humans undergoing endovascular procedures.

## Background

Contrast-induced nephropathy (CIN) is the commonest cause of iatrogenic renal injury and its incidence is increasing due to the rise in the number and complexity of endovascular interventions [1]. This is increasingly apparent with prolonged endovascular aortic interventions such as fenestrated endovascular aortic aneurysm repair where high intra-arterial x-ray contrast media volumes may be administered. CIN is therefore a significant clinical problem, accounting for 10% of all hospital-acquired renal insufficiency [2], and is strongly associated with adverse clinical outcomes and prolonged hospital length of stay [3]. The development of CIN is associated with an increase in both in-hospital and 1-year mortality, irrespective of whether dialysis is necessary [4]. The quoted incidence of renal insufficiency following open and endovascular aneurysm repair (EVAR) varies widely [1, 5, 6], and it is clear that the cause of this damage is complex and may include aortic/stent-graft manipulation, blood loss and anaesthetic factors as well as CIN. In spite of these complications the patient benefit of less invasive EVAR (compared to conventional surgery) is, however, significant in that the risk of renal hypo perfusion secondary to hemodynamic instability and cross clamping is eliminated, surgical trauma is reduced, and ischemia-reperfusion injury is attenuated. However, EVAR still causes a significant systemic reaction, possibly through a combination of ischemia-reperfusion injury. Moreover, while the contemporary literature may suggest that EVAR protects the kidneys perioperatively, emerging data raise the possibility that long-term renal injury may be greater following EVAR than after an open operation [1].

Much attention has been paid to measures to reduce the incidence and severity of renal insufficiency associated with CIN. A recent meta-analysis [7] investigating multiple agents in this setting including N-acetylcysteine (NAC), theophylline, furosemide, dopamine, bicarbonate, iloprost and statins concluded that only NAC and theophylline had a demonstrable benefit compared with hydration alone, whereas furosemide had a detrimental effect upon renal function. Within the setting of vascular surgery the role of NAC is controversial [8–10] and not universally utilised. The role of AA in CIN associated with percutaneous cardiac interventions and radiographic imaging, likely due to its antioxidant properties, has been previously demonstrated to be beneficial in high-risk patients [10, 11] however the evidence for its routine use is again unclear [12, 13].

The mechanism of CIN is not well established but is thought to be related to renal vasoconstriction and increased osmotic load producing regional hypoxia, particularly in the renal medulla, which is susceptible to hypoxia. Post-ischaemic oxidative changes lead to an increase in production of free radical species, which in turn produce renal damage [14, 15]. Therefore AA as an anti-oxidant is thought to work in its role as a free radical scavenger.

Currently there is no data from either clinical or in vivo models assessing the potential of AA as a nephroprotective agent in patients undergoing endovascular aneurysm repair. The aim of this study was to investigate the role of AA in preventing CIN using an in vivo murine model. This was achieved using a variety of biomarkers including NGAL, RBP-4, apoptosis (TUNEL and Caspase 3) and karyolysis/inflammation (H&E staining) known or suspected to be associated with kidney damage. The biomarkers were measured using different assay formats (immunohistochemistry, ELISA, RT-PCR and histopathology) in order to determine if pre-treatment with AA has a potential therapeutic benefit in preventing CIN.

## Methods

Charles River UK performed in vivo studies in which four groups of six BALB/c mice (weighing 6g each) were injected with nitric oxide synthase inhibitors (N^G^ -nitro-L-arginine methyl ester, 10mg/kg) and an inhibitor of prostaglandin synthesis (indomethacin 10mg/kg) intraperitoneally before Omnipaque administration (isohexol, 350mg iodine/ml, 1.5–3g iodine/kg). This is an established model for generating reproducible renal failure following radiocontrast injection [16]. Two groups were then given either low (0.056g/kg) or high dose AA (0.112g/kg) prior to contrast administration then after 12 and 24 h. The positive control group was given intravenous saline hydration 24 h post-administration of contrast media. The negative control group comprised no administration of contrast media and received intravenous saline hydration alone for the same time period. Urine was collected and pooled from each group before and after contrast injection, at 48 and 96 h then centrifuged and analysed for NGAL and creatinine. The NGAL: creatinine ratio was then calculated. At the end of the study kidneys from each mouse were explanted and frozen at -80°C.

### Histopathology

Standard histological techniques were used to fix and embed one kidney, per mouse, in paraffin. Representative slices were stained with haematoxylin-eosin (HE) and scored for damage to the tubules. The parameters for scoring were karyolysis (scored in both the inner and outer cortex) and inflammation (as scored by the presence of mononuclear cells in both the cortex and medulla). Each parameter was scored from 0 to 4 with 0 representing no damage, 1 as mild, 2 as moderate, 3 as severe and 4 as very severe/extensive.

#### Immunohistochemistry and imaging

The explanted murine kidneys were frozen in liquid nitrogen and one kidney from each animal used for Caspase-3 and TUNEL (transferase mediated dUTP nick –end labelling). The tissue was cut to 10-μm sections on cryostat and thaw mounted on SuperFrost slides (VWR, West Sussex, UK). For both detection methods sections were fixed in 2% paraformaldehyde (PFA) in 1x phosphate buffered saline (PBS) for 10min and rehydrated in 1x PBS for 15min. For activated Caspase-3 staining only cell membranes were permeabilised in PBS containing 0.5% Tween-20 for 10min and washed 3x with 1x PBS + 0.05% Tween-20 for 5 min each. The sections were blocked for 1h in blocking solution containing 2% BSA and 5% goat serum in 1x PBS and washed 3x with 1x PBS for 5 min each. All incubation steps were performed at room temperature. Rabbit polyclonal anti-active Caspase-3 (p17 fragment) antibody (Abcam, Cambridge, UK) was diluted 1:100 in blocking solution and incubated with the sections overnight at 4°C. After 3 washes in 1x PBS Alexa Fluor 488-labeled goat anti-rabbit secondary antibody (Invitrogen. Paisley, UK) was applied at 1:100 dilution in blocking solution and incubated for 1h at room temperature in the dark. After 3 washes in 1xPBS/0.05% Tween20 the sections were mounted with Vectashield medium containing DAPI (Vector Laboratories, Peterborough, UK).

For TUNEL staining APO-BrdU™ TUNEL Assay Kit (Invitrogen, Paisley, UK) was used and protocol adapted for fluorescence microscopy. After fixation and rehydration as described above, sections were incubated in 70% ice-cold ethanol at -20°C for 30 min. Ethanol was removed and rinsed 3x with Wash Buffer. DNA-labelling solution was prepared as per manufacturer instructions and applied on sections overnight at 4°C. After washing with Rinse Buffer Alexa Fluor®488-labeled anti-BrdU mouse monoclonal antibody was applied as per manufacturer instructions and incubated 1h at room temperature in the dark. Sections were rinsed with PBS/0.05% Tween and stained with propidium iodide/RNase A staining buffer for 1 h at room temperature in the dark. After 3 washes in 1xPBS/0.05% Tween20 the sections were mounted with Vectashield medium containing DAPI (Vector Laboratories, Peterborough, UK).

Confocal fluorescent images were acquired using Zeiss LSM 510 Meta Confocal system (LSM software release 3.2) coupled to a Zeiss Axiovert 200 microscope. All images were acquired at room temperature.

#### mRNA analysis by quantitative real-time PCR

Quantitative real-time PCR (qRT-PCR) was conducted on the explanted mouse kidneys. Total RNA was extracted from frozen tissue samples using PARIS™ Kit (Life Technologies – Invitrogen, Paisley, UK). First strand cDNA synthesis was performed using QuantiTect Reverse Transcription Kit (Qiagen, Manchester, UK) from 100ng total RNA. All measurements were in duplicate and the relative expression levels of lipocalin-2 (Lcn-2) and retinol binding protein-4 (RBP-4) were each normalized hypoxanthine-guanine phosphoribosyltransferase (HPRT), glyceraldehyde 3-phosphate dehydrogenase (GAPDH) and beta-2 microglobulin (B2M). qRT-PCR was performed on a BioRad CFX96 Real time PCR machine using QuantiTect SYBR Green PCR Master Mix (Qiagen, Manchester, UK) and validated primer pairs Quantitact Primer Assay (Qiagen, Manchester, UK) for all tested and housekeeping genes. The PCR efficiency for each set of primers was previously established by analysing serial dilutions of cDNA.

#### Biochemical analysis of NGAL

NGAL was measured using a two-site time resolved fluorescence DELFIA immunoassay (PerkinElmer Inc, MA, USA). Nunc Maxisorp plates (PerkinElmer Inc, MA, USA) were coated overnight with a monoclonal anti-NGAL antibody (R&D Systems, MN, USA). After coating, the plates were washed three times with DELFIA wash buffer and blocked with 300μl of 1% BSA in PBS. Urine samples were diluted 1 in 5 in DELFIA Multibuffer (PerkinElmer Inc, MA, USA) before analysis. Standards were prepared from recombinant murine NGAL (R&D Systems, MN, USA). 50μl standards, diluted urine or diluted QC samples were added to the plate in duplicate followed by 100μl DELFIA Multibuffer (PerkinElmer Inc, MA, USA). The plate was incubated at room temperature for 2 h on a plate shaker. The plate was then washed 4 times using an automated plate washer and a biotinylated polyclonal anti-NGAL detection antibody (R&D Systems, MN, USA) added to the wells. The plate was then incubated at room temperature for a further 2 h on a plate shaker. The plate was then washed 4 times using an automated plate washer and Europium labelled streptavidin (PerkinElmer Inc, MA, USA) added to the wells. The plate was incubated at room temperature for a further 40 min on a plate shaker. The plate was then washed 6 times using an automated plate washer and 200μl Enhancement solution (PerkinElmer Inc, MA, USA) added to the wells. The plate was incubated for 10 min on a plate shaker before time-resolved fluorescence readings were taken on a Victor 3 plate reader (PerkinElmer Inc, MA, USA). Results were calculated using the MultiCalc software package (PerkinElmer Inc, MA, USA). The assay range was 78.1 pg/ml to 5000 pg/ml. Creatinine was measured using a standard certified biochemistry assay (Department of Biochemistry, Cambridge University NHS Trust).

For NGAL analysis from explanted kidney lysates, individual kidneys were ground in liquid nitrogen and processed using a PARIS™ kit (Life Technologies – Invitrogen, Paisley, UK); half the sample was used for total protein analysis. Total protein was estimated using a Bradford assay (Thermo Scientific, Loughborough, UK) and each kidney sample was diluted to 20 μg protein/ml. NGAL concentration was determined using a Human Lipocalin-2/NGAL Quantikine ELISA Kit (R&D Systems, Abingdon, UK).

#### Statistics

Statistical analysis was undertaken using GraphPad Prism v5 (GraphPad Software, La Jolla, CA). Binomial data were analysed using contingency table analysis and Fisher’s exact test used to calculate significance levels. Continuous data was analysed with students’ *t*-test. Significance was determined using 95% confidence (*p* < 0.05) in all cases.

## Results

**Fig. 1:**
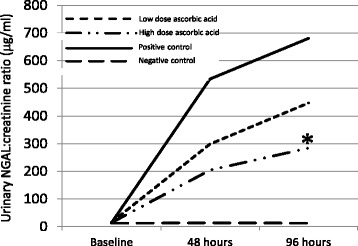
Mean urinary NGAL:creatinine ratio (ug/ml) at 0 (baseline), 48 and 96 h following administration of contrast media between AA treated groups (high and low dose AA). Significant (*p* < 0.05) changes in NGAL:creatinine ratio are indicated (*) compared to the positive control group

Urinary NGAL:creatinine ratio was significantly lower in both the high and low dose AA groups when compared to the positive control (saline hydration 24 h post-contrast administration) group (Fig. 1). In the low dose AA group there was a 44% reduction in mean NGAL:creatinine ratio at 48 h compared to the positive control group (298.4μg/mmol versus 533.6μg/mmol, respectively). The reduction in mean NGAL:creatinine urine ratio appeared to persist at 96 h although this had reduced to 34% compared to the positive control group. It was apparent that there was a reasonably high degree of variability in the mean NGAL:creatinine ratios observed for each group of mice and this was reflected by a lack of significance at both time points for the low dose AA group. A greater (62–58% reduction over 48–96 h time period) and significant (*p* = 0.049) reduction in the mean NGAL:creatinine ratio was however observed with the high dose AA group at 96 h

### Histopathology and immunohistochemistry

**Fig. 2:**
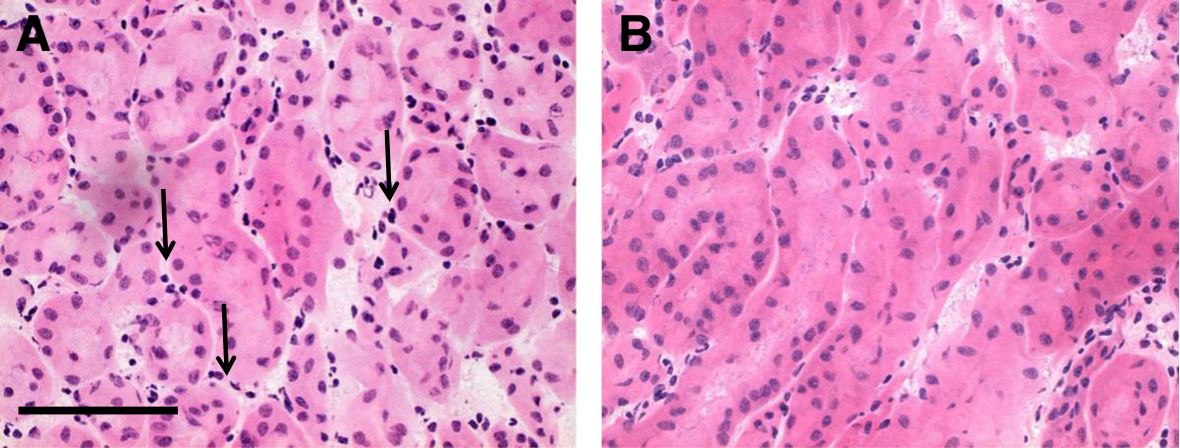
Representative histopathological examination of paraffin embedded explanted kidneys using haematoxylin-eosin staining observed at x400. **a** - Histology from positive control (group 3) showing karyolysis and karyorrhexis in the renal cortical tubular epithelium. The sections showed a clear interstitial infiltrate of mononuclear cells (arrows). **b** - Histology from high dose AA treatment (group 2) demonstrating fewer infiltrating mononuclear cells. Scale bar represents 50μm

Explanted kidneys from the four groups of mice were analysed by histology and immunohistochemistry for inflammatory and apoptotic markers. Histological analysis of paraffin sections using haematoxylin and eosin staining of explanted kidneys revealed evidence of reduced inflammation through mononuclear cell infiltration with both the high dose and low dose AA treatments (Table 1 and Fig. 2). The positive control also showed higher levels of karyohexis in the renal cortical tubular epithelium compared to both AA treated groups, although there was no difference between any of the groups in the level of karyolysis (data not shown).

**Table 1:** Histopathology (haematoxylin-eosin) scoring of explanted kidneys for mononuclear inflammatory cell infiltration from four groups at 400x magnification where the groups have been scored: 0 = no damage, 1 = mild, 2 = moderate, 3 = severe and 4 = very severe/extensive

Test group	Individual replicate mouse kidney		
	1	2	3
Group 1 – Low dose AA	1+ cortex 0 medulla	1+ cortex medulla not present	1+ cortex medulla traumatised
Group 2 – High dose AA	1+ cortex 0 medulla	2+ cortex 0 medulla	1+ cortex medulla not present
Group 3 – Positive Control	3+ cortex 0 medulla	3+ cortex very little medulla present	3+ cortex and neutrophils 1+ medulla and neutrophils
Group 4 – Negative Control	2+ cortex 1+ medulla	2+ cortex 2+ medulla	2+ cortex 1+ medulla

**Fig. 3:**
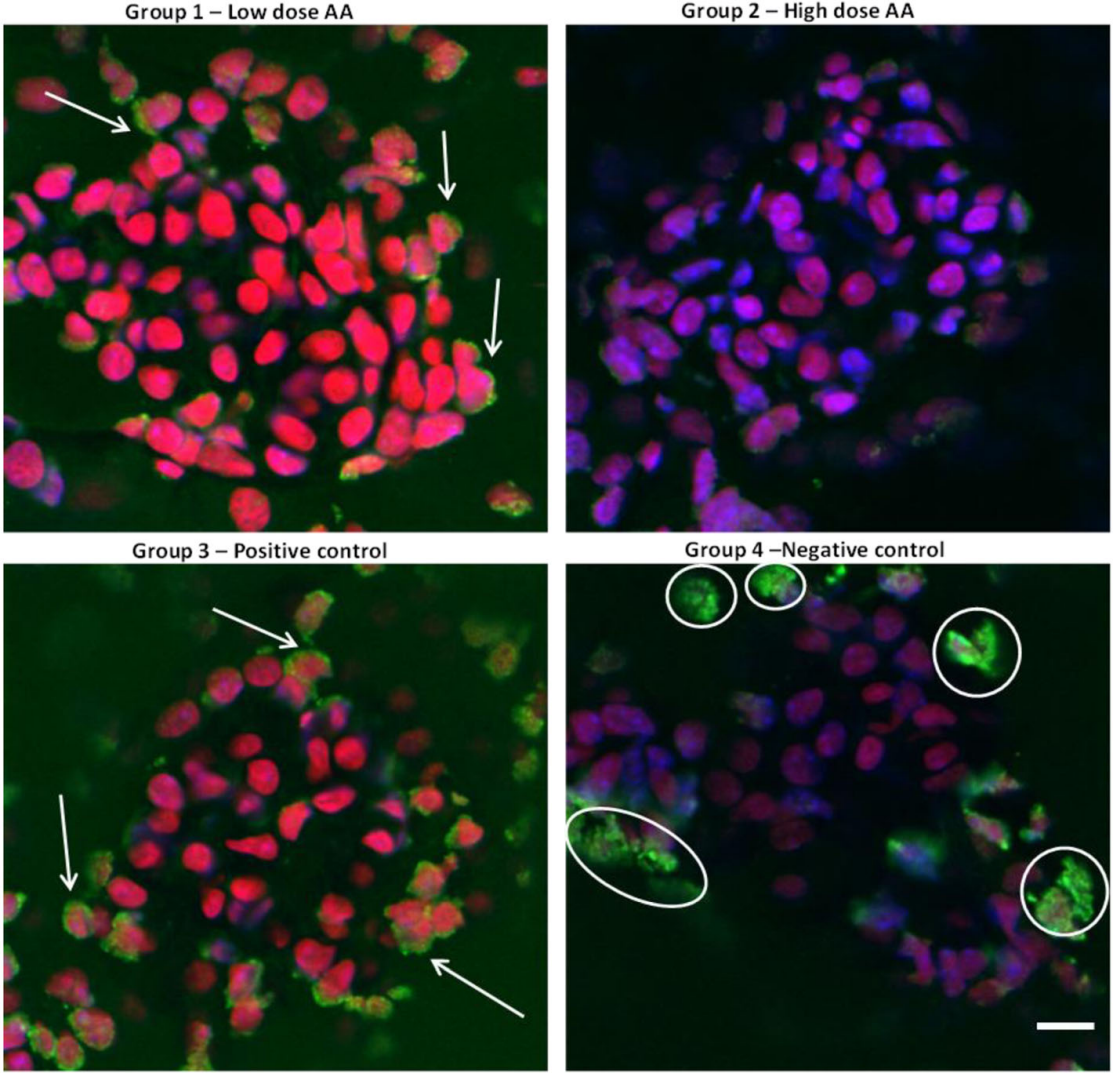
TUNEL immunohistochemistry of kidney glomeruli. DNA nicking Brdu staining (green fluorescence and indicated by arrows) and DAPI counterstained nuclei (red). Note the negative control showed high levels of non-specific staining indicated by white circles. Scale bar represents 10μm

**Fig. 4:**
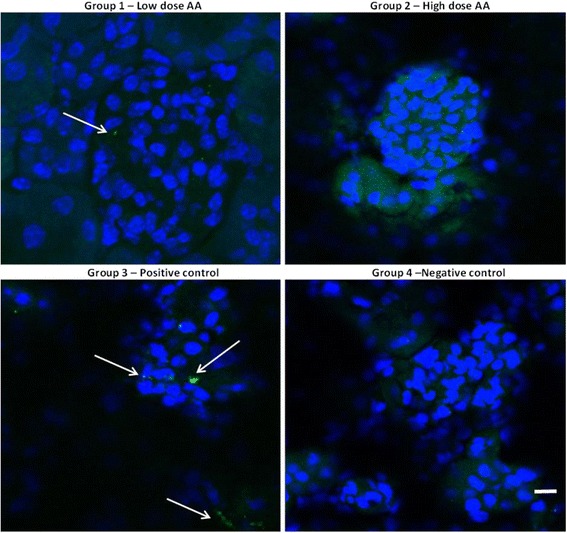
Caspase-3 immunohistochemistry of renal glomeruli and tubular cells. Activated caspase-3 (green fluorescence indicated by arrows) and DAPI counterstained nuclei (blue). Scale bar represents 10μm

Immunohistochemical analysis of frozen kidney sections was performed by examining changes in the glomeruli and renal tubules of the explanted kidneys, where treatment with contrast media is known to induce renal cell apoptosis [17]. The TUNEL assay was used to determine the level of apoptosis and the results showed progressively lower levels of DNA nicking (TUNEL assay) in the low and high dose AA groups compared to the positive control group (Fig. 3). Further confirmation of the TUNEL assay data was obtained by immunohistochemical analysis for activated Caspase-3 expression. Caspase-3 activation occurs upstream of DNA nicking, thus early stages of induction of apoptotic signalling could be determined by measuring activated Caspase-3 (using an antibody that specifically recognises the p17 fragment of the active enzyme) in the kidney sections. Although Caspase-3 staining was relatively weak compared to the TUNEL assay the results across the sections were consistent with the results obtained in the TUNEL assay, in that, a reduction in the Caspase-3 expression in mice treated with AA was observed (Fig. 4). Importantly, Caspase-3 expression appeared to correlate with the dose of AA i.e. the high dose treatment resulted in the largest reduction in Caspase-3 expression. Both the histo- and immunohistochemical analysis of kidneys from the AA treated mice showed a reduction in the level of inflammation and a reduction in both early and late stage apoptosis. Overall the reduction in inflammation and apoptosis appeared be greater with the high dose AA treatment.

### Biochemical analysis NGAL, Lcn-2 and RBP-4

**Fig. 5:**
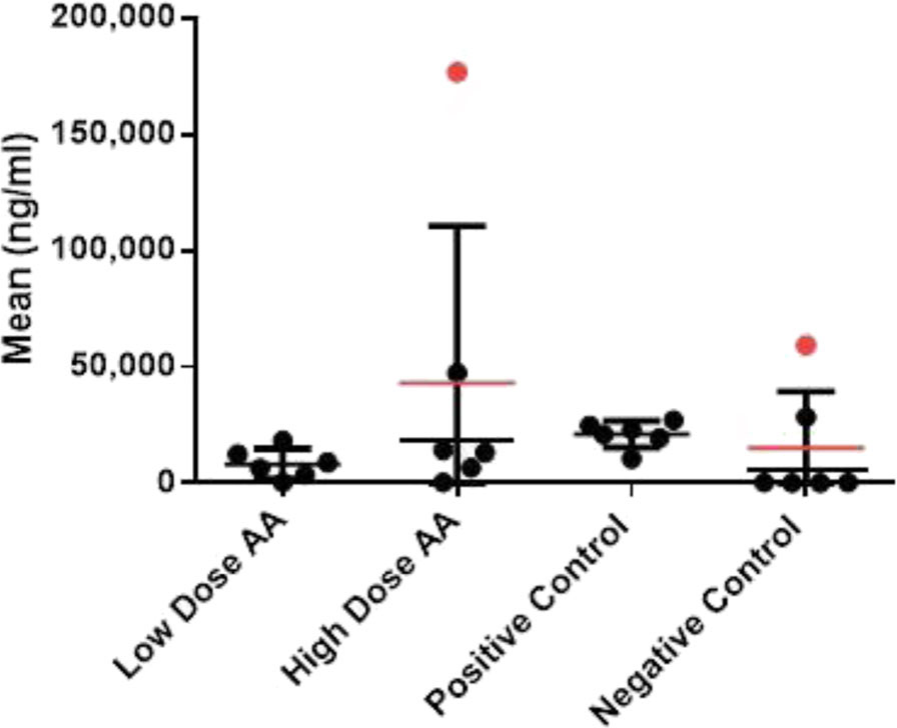
NGAL ELISA of kidney lysates for individual mice. Dot plot showing NGAL expression in individual mice (ng/ml) the mean (horizontal lines) and range (SD) for all 6 mice in each group are shown. Outliers (excluded from analysis) are indicated by red circles and mean values represented by horizontal lines are for entire group (red line) or for group excluding outlier data (black line). The SD is calculated for all data including outliers

Additional molecular and biochemical analysis of the explanted kidneys in which biomarkers for NGAL, Lcn-2 and RBP-4 was performed to determine the effect of AA treatment on CIN. Kidney lysates were analysed by NGAL-specific ELISA (Fig. 5) and the results showed that treatment with AA significantly (*p* < 0.05) reduced NGAL expression in the low dose AA group by 57% compared to the positive control group (from 20,948 ng/ml to 8,315ng/ml). The high dose AA group failed to show a reduction, however it was clear that ELISA data from two mice (one in the high dose AA group and one in the negative control) where extremely high resulting in very large SD’s (indicated by data labels in Fig. 5). Both data sets from both mice were considered erroneous and therefore excluded from subsequent analysis. Removal of the two outliers in the data analysis reduced the SD and resulted in a reduction in NGAL expression in the high dose AA group of 22% compared to the positive control (from 20,948 ng/ml to 16,407 ng/ml) although this was not significant (*p* = 0.4409).

**Fig. 6:**
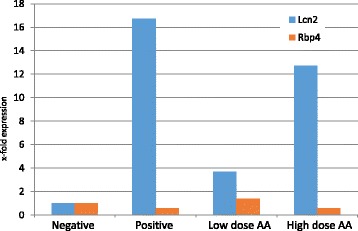
Gene transcript analysis of Lcn-2 and RBP-4 by qRT-PCR normalised to HPRT, GAPDH and B2M in the positive and negative controls versus the two treatment arms. Data is shown as fold increase above the negative control

To further elucidate whether the difference in high and low dose NGAL expression was genuine qRT-PCR analysis of Lcn-2 gene (encoding NGAL protein) mRNA transcript expression in mRNA extracted from mouse kidneys was performed (Fig. 6). A significant (*p* < 0.05) decrease in Lcn-2 transcript was observed in the low dose AA group compared to the positive control (16.75 fold expression vs. 3.69 fold expression relative to the negative control group). However when comparing high and low dose AA groups the high dose group had a higher level of Lcn-2 transcript compared to the low dose AA group (12.74 versus 3.69 fold expression relative to negative control, respectively). The level of Lcn-2 transcript was however still significantly lower in the high dose AA group compared to the positive control. Thus the results of the NGAL ELISA and Lcn-2 mRNA data correlate and the unexpected finding that the greatest reduction in NGAL expression was observed in the low dose AA group was confirmed..In order to assess the integrity of the proximal renal tubule additional analysis by qRT-PCR of RBP-4 transcript was performed (Fig. 6). Measurement of RBP-4 in urine is arguably the most sensitive biomarker for loss of function of the human proximal renal tubule [18] although analysis of transcripts from kidney mRNA extracts revealed no changes in the levels of expression between the four groups.

## Discussion

Contrast induced nephropathy is a predominant cause of hospital acquired renal injury. There are an increasing number of both diagnostic investigations and therapeutic procedures performed using radio-contrast. Furthermore an ageing population, with a higher incidence of underlying renal impairment, make it imperative that greater attention is focused on the aetiology of CIN in order to formulate effective prophylactic and therapeutic strategies to reduce its incidence and associated morbidity and mortality [19]. Contemporary evidence has highlighted significantly higher mortality rates in patients who suffer acute kidney injury after emergency aneurysm surgery at both 30-days and 12-months [20]. Clearly there is a clinical need for therapeutic strategies aimed at prevention of CIN, and in this study we focused on investigating the potential of ascorbic acid in a murine model.

An established [16] murine model was selected that provides an effective model for CIN, and where a series of biomarkers could be measured to determine the nephroprotective effect of high dose AA on renal toxicity. Key biomarkers, from urine and explanted kidneys, selected for this study included NGAL (and the corresponding Lcn-2 mRNA transcript), NGAL:creatinine ratio (urine), apoptotic markers and kidney histology. Overall, the results demonstrated compelling evidence that in this murine model AA functions as a nephroprotective agent against CIN. These results add further weight to the evidence from the percutaneous cardiology setting [10, 12] that AA may be an agent, which could be transferred to the endovascular setting to counteract the influence of contrast media.

All the animals in this murine model had normal renal function prior to contrast administration. We know that in human studies patients with normal renal function are at the lowest risk of developing CIN. Further work in a murine model with pre-existing renal failure may demonstrate greater benefits of AA. The risk of developing CIN is greatest in patients with pre-existing renal impairment and diabetes. These patients are the most likely to benefit from the use of a nephroprotective agent. The risk of CIN in patients with normal renal function is likely to increase with the increasing complexity of endovascular interventions.

The low dose of AA trialled in this study was from a clinical perspective considered a ‘mega-dose’ in that the standard prophylactic daily dose quoted by the British National Formulary is 25–75mg (with a therapeutic dose of 250mg), therefore the high dose group should be regarded a high ‘mega-dose’. No deleterious effects either high or low dose AA were detected in this study, and both doses conferred some level of nephroprotection against CIN although in both the urinary NGAL:creatinine ratio and apoptosis assays, the high dose AA group appeared to be most resistant to CIN. Surprisingly, there have been no human clinical trials investigating the use of such a high dose AA in this setting, even used as a very short treatment. There have been advocates for mega-dosing of AA, particularly surrounding alternative medicine including the treatment of the common cold and in cancer therapy. The side effects from these doses (typically 2–4g daily) are nausea, vomiting, diarrhoea and renal calculi. However previous research has suggested that high dose AA is safe; Spargias et al. [10] used mega-doses of AA, administering 3g followed by 2g and 2g, and observed no negative side effects in their study group. Our study design calculated the low AA dose administered to the mice to be equivalent to the human dose utilised by Spargias et al [10]. The dose of AA was based upon the standard human dose per kilogram body weight, with the high dose representing twice this dose, however still remaining within the previous documented megadosing regimens.

The most frequently quoted definition of CIN is an increase in serum creatinine of 25–50% from baseline, generally occurring within 24 h of contrast administration. With this in mind urine collection was performed at baseline, 48 and 96 h followed by sacrifice of the mouse, in order to maximise the observed level of renal damage following contrast administration. Serum creatinine however, is relatively insensitive only rising out of the normal range when 50% of the functioning renal mass is lost [19]. Furthermore even modest changes in serum creatinine have a strong association with in-hospital mortality [21]. Subclinical markers of kidney injury, such as, NGAL, retinol binding protein and albumin/creatinine ratios, not only allow the identification of renal damage that is not normally identified by creatinine measurement alone, but also they can potentially can identify the beneficial impact of therapies aimed at mitigating the effects of radio-contrast media. Furthermore, the evaluation of peri-operative renal function and the impact of therapeutic strategies have been hampered by the lack of sensitive and specific biomarkers of acute kidney injury. We have previously shown the value of retinol binding protein (RBP) as a reliable biomarker of CIN [8, 22]. In more contemporary work we have also demonstrated the potential of NGAL to identify early renal injury in patients undergoing endovascular AAA repair [23]. In this study, the positive control data from the murine model confirmed observations in previous studies [17, 24, 25] where it was demonstrated that apoptosis triggered by CIN resulted in a higher frequency of apoptotic cells in the glomerulus and renal tubules. This supports the notion that at least one potential mechanism of CIN in patients may involve caspase-dependent apoptosis, moreover administration of high dose AA appears to reduce apoptosis in the glomerulus.. There also appears to be a dose-response relationship with regard the effect of AA administration on apoptosis. One unexpected finding was the fact that the low dose AA appeared to have a more potent effect in reducing NGAL expression (as both NGAL protein and Lcn-2 transcript) in the kidneys. There is no clear explanation for this result although differences in the dose groups may be due to the kinetics of NGAL induction (which were not investigated in this study), thus the timing of the kidney removal after treatment with contrast media may explain this finding. It is clear that these preliminary observations offer potential insights into the mechanisms of CIN and possible treatments to ameliorate nephrotoxicity although further work is required in both animal models and patients to understand optimal dosing of AA and to investigate the effects of AA in reducing oxidative stress in kidneys. One of the limitation of this study is that biomarkers of oxidative stress were not measured and this may have thrown further light on the mechanism of action of ascorbic acid in reducing CIN.

The concerns regarding CIN in EVAR patients are not just related to the index procedure. There is increasing evidence of long-term renal dysfunction that maybe related in part to further contrast administration during surveillance CT imaging or at the time of subsequent reinterventions. Measures to ameliorate CIN should be taken during any subsequent contrast administration in higher risk EVAR patients. There is now good contemporary data confirming that AA reduces the incidence of CIN in patients undergoing coronary angiography by up to a third [26]. One would anticipate that a similar benefit would be observed in patients undergoing peripheral vascular and aortic interventions. High dose AA appears to be well tolerated in the coronary population and the data presented in this study would support a clinical trial of AA in patients undergoing non-coronary endovascular interventions.

## Conclusion

In conclusion this study presents comprehensive basic science evidence for the role of AA as a nephroprotective agent following contrast administration. Further work is warranted to establish the role of this therapy in the prevention of CIN in humans undergoing endovascular procedures.
